# Unified Deep Learning-Based Mouse Brain MR Segmentation: Template-Based Individual Brain Positron Emission Tomography Volumes-of-Interest Generation Without Spatial Normalization in Mouse Alzheimer Model

**DOI:** 10.3389/fnagi.2022.807903

**Published:** 2022-03-04

**Authors:** Seung Yeon Seo, Soo-Jong Kim, Jungsu S. Oh, Jinwha Chung, Seog-Young Kim, Seung Jun Oh, Segyeong Joo, Jae Seung Kim

**Affiliations:** ^1^Department of Nuclear Medicine, Asan Medical Center, University of Ulsan College of Medicine, Songpa-gu, South Korea; ^2^Department of Biomedical Engineering, Asan Medical Center, University of Ulsan College of Medicine, Songpa-gu, South Korea; ^3^Department of Health Sciences and Technology, Samsung Advanced Institute for Health Sciences & Technology (SAIHST), Sungkyunkwan University, Songpa-gu, South Korea; ^4^Department of Intelligent Precision Healthcare Convergence, Sungkyunkwan University, Suwon-si, South Korea; ^5^Department of Convergence Medicine, Asan Medical Center, University of Ulsan College of Medicine, Songpa-gu, South Korea

**Keywords:** mouse brain, deep convolutional-neural-network (CNN), inverse-spatial-normalization (iSN), skull-stripping, template-based volume of interest (VOI)

## Abstract

Although skull-stripping and brain region segmentation are essential for precise quantitative analysis of positron emission tomography (PET) of mouse brains, deep learning (DL)-based unified solutions, particularly for spatial normalization (SN), have posed a challenging problem in DL-based image processing. In this study, we propose an approach based on DL to resolve these issues. We generated both skull-stripping masks and individual brain-specific volumes-of-interest (VOIs—cortex, hippocampus, striatum, thalamus, and cerebellum) based on inverse spatial normalization (iSN) and deep convolutional neural network (deep CNN) models. We applied the proposed methods to mutated amyloid precursor protein and presenilin-1 mouse model of Alzheimer’s disease. Eighteen mice underwent T2-weighted MRI and ^18^F FDG PET scans two times, before and after the administration of human immunoglobulin or antibody-based treatments. For training the CNN, manually traced brain masks and iSN-based target VOIs were used as the label. We compared our CNN-based VOIs with conventional (template-based) VOIs in terms of the correlation of standardized uptake value ratio (SUVR) by both methods and two-sample *t*-tests of SUVR % changes in target VOIs before and after treatment. Our deep CNN-based method successfully generated brain parenchyma mask and target VOIs, which shows no significant difference from conventional VOI methods in SUVR correlation analysis, thus establishing methods of template-based VOI without SN.

## Introduction

^18^F-fluorodeoxyglucose positron emission tomography (^18^F-FDG PET) is a useful imaging technique that enables the investigation of glucose metabolism-based functional imaging not only in human brains but also in mouse brains (Som et al., 1980; Bascunana et al., 2019).

Because mouse brains have different shapes and sizes, spatial normalization (SN) of individual brain PET and/or magnetic resonance imaging (MRI) onto standard anatomical spaces is required for objective statistical evaluation (Ma et al., 2005). Moreover, we can apply common volume-of-interest (VOI) templates for spatially normalized individual brain PET and MR images, which can be directly used without labor-intensive manual tracing and further can define individual brain-specific VOIs using inverse transformation of SN.

To do this, PET-based SN (i.e., the SN of individual PET images onto a ligand-specific PET template) can be conducted, but SN using PET alone may be vulnerable to local uptake changes, and disease-specific uptake patterns may not be optimal compared to SN based on anatomical information. Moreover, MR-based SN has been preferred to project individual brain PET and/or MR images into a template space (Ashburner and Friston, 1999; Gispert et al., 2003) because MRI is independent of changes in uptake patterns due to diseases in PET images and is advantageous in terms of anatomical precision.

In human brain studies, many neuroimage analysis tools including statistical parametric mapping (SPM), FMRIB Software Library (FSL—Woolrich et al., 2009; Jenkinson et al., 2012), and Elastix (Klein et al., 2010; Shamonin et al., 2014) have been widely used to perform SN. However, there are limitations in the use of these tools in mouse brain research due to various differences between human and mouse brains, such as the scale, shape, and image contrast (including distribution of gray matter).

In some human brain studies, the template used for SN was skull-stripped to avoid potential spatial misregistration due to soft tissues around the skull and brain (Acosta-Cabronero et al., 2008). Because most mouse brain templates have been based on skull-stripped images, skull-stripping has been considered a prerequisite for the SN of mouse brain MRI and/or PET images (Fein et al., 2006; Feo and Giove, 2019).

Template-based (also known as atlas-based) brain skull-stripping and brain VOI segmentation methods in mouse brain MRIs have shown accurate segmentation performance in prior studies (Nie and Shen, 2013; Feo and Giove, 2019). In these studies, PET or MRI images of individual brains were registered onto predefined templates of average mouse brains using affine transformations, occasionally followed by non-linear registration for more precise registration of the individual brains onto a brain template. Subsequently, the template brain skull-stripping results were used as starting (or seeding) points to define the individual brain skull-stripping and segmentation. Although these methods were relatively accurate, most of them were applied semiautomatically, not only resulting in SN with reduced reliability due to inter- and intrarater reliability issues (Yasuno et al., 2002; Kuhn et al., 2014) but also involving a time-consuming process. In addition, degradation of image quality may occur (in preserving the integrity of the original voxel intensities), because SN requires spatial transformation of the images onto a template, which, in turn, requires image intensity interpolation.

Recently, several deep learning (DL)-based skull-stripping methods have been studied. Hsu et al. (2020) devised a DL-based framework to automatically identify mouse brains in MR images. The brain mask was generated using MR image patches randomly cropped as input of the 2D U-Net architecture. For the evaluation of the model, a manually traced brain mask by an anatomical expert and a brain mask generated by the model were evaluated by Dice coefficient and Jaccard index and also positive predictive value and sensitivity, and the results showed better performance than conventional methods (Chou et al., 2011; Oguz et al., 2014; Liu et al., 2020). Similarly, De Feo et al. (2021) proposed multitask U-Net (MU-Net) to accomplish skull-stripping and region segmentation. 128 T2 MR image from 32 mice at 4 different ages were used as inputs to perform the training and validation of their proposed model, and five manually traced regions consisting of cortical, hippocampus, ventricular, striatum, and brain masks were used as labels. They demonstrated that MU-Net was able to effectively reduce the inter- and intrarater variability that occurs when skull-stripping and region segmentation are performed manually and showed better performance than the latest multiatlas segmentation methods (Jorge Cardoso et al., 2013; Ma et al., 2014).

By contrast, DL-based SN has been a very challenging problem involving many DL-based medical image preprocessing steps. Recently, an interesting DL-based “pseudo” PET template generation method (as the preprocessing of amyloid PET SN) has been developed using a convolutional autoencoder and a generative adversarial network (Kang et al., 2018). Nonetheless, this class of methods commonly generates a “pseudo” linearly or non-linearly registered individual brain onto the template using a convolutional neural network (CNN), instead of estimating the actual spatial transformation of the individual images onto the template, inevitably requiring additional efforts for the final SN. Another class of CNN-based spatial registration methods has been developed, called spatial transformer networks (STNs, Jaderberg et al., 2015). However, most of them are demonstrated as 2D registration and linear (rigid body or affine) transformation tools. Although a diffeomorphic transformer network was recently developed by Detlefsen et al. (2018) using a continuous piecewise affine-based transformation, its validation study as an SN tool for PET or MR images has not been conducted thus far. Notably, a DL model-based SN of Tau PET has been developed, which repeatedly estimates the sets of rigid and affine transformations using a CNN (Alvén et al., 2019). Although rigid and affine transformations have been implemented and evaluated, a non-rigid deformation has not been. Moreover, this class of methods requires a complicated CNN architecture consisting of cascades of many regression and spatial transformer layers, that is, numerous CNN parameters to be estimated. This reduces the clinical feasibility of these methods in that considerable amounts of data are required for effective training. Taken together, thus far, the SN problem in isolation has not been fully resolved, even in recent DL-based literature, including not only image segmentation (Delzescaux et al., 2010; Bai et al., 2012; Nie and Shen, 2013; Feo and Giove, 2019) but also apparently more challenging image generation methods, such as PET-based MR generation, to support SN (Choi et al., 2018). Considering that DL-based image generation can resolve these challenging issues of medical image preprocessing, we were motivated to reformulate the challenging SN problem into a more easily tractable image generation problem of VOI segmentation. To bridge SN and segmentation, we used the useful approach of iSN.

The iSN process involves the operation of generating the inverse of a deformation field and resampling a spatially normalized image back to the original space (i.e., an individual brain space—Ashburner et al., 2000). Indeed, we can generate inversely normalized VOIs (iVOIs) in an individual brain space by applying the iSN technique to VOI templates, as performed in many neuroimaging studies. Representatively, neuroimage analysis tools such as MarsBar and the deformation toolbox of SPM conduct iSN-based VOI analysis in an individual (native) brain space and have been frequently used in many brain PET quantification studies (Kim et al., 2010, 2015; Cho et al., 2014). Specifically, computed tomography (CT)-based SN was used for ^18^F-fluoro-propyl-carbomethoxy-iodophenyl-tropane (^18^F-FP-CIT) PET analysis using an inverse-transformed automatic anatomical label (AAL) VOI template in the two previous studies (Cho et al., 2014; Kim et al., 2015). Similarly, iVOIs were defined using the iSN of the Brodmann area or callosal template VOIs as the seed points for diffusion tensor tractography (Oh et al., 2007, 2009). Although attenuation correction (AC) can be conducted directly using CT transmission data in conventional PET/CT, it is difficult to conduct AC in PET/MR. To resolve this issue, several studies have employed template-based AC (Hofmann et al., 2009, 2011; Wollenweber et al., 2013; Sekine et al., 2016). In particular, Sekine et al. (2016) projected an atlas-based pseudo-CT to a patient-specific PET/MR space using iSN methods, which generated a single-head atlas from multiple CT head images.

Inspired by these concepts, we developed a new method for generating the iVOI labels of a deep CNN in an individual brain space. Consequently, we could reduce the abovementioned complicated problem of SN for the final VOI quantification into a much simpler problem of VOI segmentation in an individual brain space that is straightforwardly tractable for modern deep CNNs such as U-Net.

Recently, individual brain-specific VOI generation methods such as the FreeSurfer software (Lehmann et al., 2010), which can produce highly concordant VOIs with respect to manually traced ground-truth VOIs, have been frequently used in many neuroimaging studies. By contrast, template-based VOI approaches such as SPM present better or equal performance than individual brain space VOIs in terms of test–retest reproducibility (Palumbo et al., 2019). Considering this information, our previous iSN-based template VOI defined in the individual brain space leverages the strengths of both methods. Employing the iSN method, we can avoid image deformation, whose magnitude can be described using the Jacobian determinant of the deformation fields (Ashburner and Friston, 2000), which can lead to differences between the effective voxel sizes of PET images in individual brain and template spaces.

In this regard, herein, we propose a unified deep CNN framework designed to conduct not only mouse brain parenchyma segmentation (i.e., skull-stripping) but also to generate target VOIs (i.e., cortex, hippocampus, striatum, thalamus, and cerebellum) in an individual brain space. This is achieved using VOI templates defined in mouse MR or PET templates without SN onto a template to facilitate automatic precision ^18^F-FDG PET analysis with MR-based iVOI using deep CNN. Consequently, we could reduce the relatively complicated brain VOI generation (by skull-stripping and SN) for precise PET quantification to a more tractable problem of DL-based iVOI segmentation in an individual brain space without conducting SN. This approach can avoid the complicated preprocessing steps for skull-stripping and SN for target VOI generation, which prevents the unwanted time-consuming process of semiautomatic skull-stripping and reduces the inter- and intrarater reliability issues. In addition, our DL-based method can perform precise PET image analysis without a network of complicated structures for SN (Kang et al., 2018; Alvén et al., 2019).

## Materials and Methods

### Data

Eighteen transgenic mice expressing an amyloid precursor protein (APP) and presenilin (PS)-1 Alzheimer’s disease (AD) mouse model underwent brain ^18^F-FDG PET and T2-weighted MR (T2 MR) imaging. The PET images were acquired by nanoScan PET/MRI 1 Tesla (nanoPM—Mediso Medical Imaging Systems, Budapest, Hungary). Eighteen mice were anesthetized with isoflurane (1.5–2%) and received an intravenous injection of ^18^F-FDG (0.15 m Ci/0.2 cc). After the acquisition of T2-weighted fast spin-echo MRI by nanoPM, the PET static images were acquired for 20 min in list mode and were reconstructed using the ordered subset maximum-likelihood algorithm using the abovementioned T2-weighted MR-based AC in the Nucline software (Reinvent Systems For Science & Discovery, Mundolsheim, France) in the following parameters—energy window: 250–750 keV; coincidence mode: 1–3; Tera-tomo3D full detector; regularization: normal; iteration x subset: 8 × 6; voxel size: 0.4 mm. The MR images were acquired using a Bruker 7.0T MRI Small Animal Scanner (Bruker, Massachusetts, United States) reconstruction options of T2 MR images as follows—repetition time: 4,500 ms; echo time: 38.52 ms; field of view (FOV): 20 mm x 20 mm; slice thickness: 0.8 mm; matrix: 256 × 256; respiration gating was applied.

### Preprocessing

All image preprocessing was performed using SPM 12 software (SPM12; Wellcome Trust Centre for Neuroimaging, London, United Kingdom) implemented in MATLAB R2018a (The MathWorks Inc.) and MRIcro (Chris Rorden, Columbia, South Carolina, United States). First, the acquired T2 MR images and ^18^F-FDG PET images were converted from DICOM format to Analyze format using MRIcro. PET images were coregistered onto MR images to match different FOVs and slice thicknesses. For brain parenchyma mask generation DL, the entire mouse brain was manually traced using in-house software (Asan Medical Center Nuclear Medicine Toolkit for Image Quantification of Excellence—ANTIQUE) (Han et al., 2016). To achieve a more precise SN of the PET image, deformation fields generated by spatial normalizing T2 MR images to the T2 MR template were applied to the corresponding PET images. In addition, to obtain a label image of the “iVOI template” (i.e., VOI template-based and iSN-based VOIs in an individual brain space) generation model, an inverse deformation field was generated and resampled from the VOI template space to the individual image space. This was achieved using the deformation toolbox of SPM, by performing inverse transformation of the deformation fields that map the individual brains onto the template brain. A quantitative evaluation of the PET images by a VOI template was used as the gold standard.

### Deep Convolutional Neural Network

**FIGURE 1:**
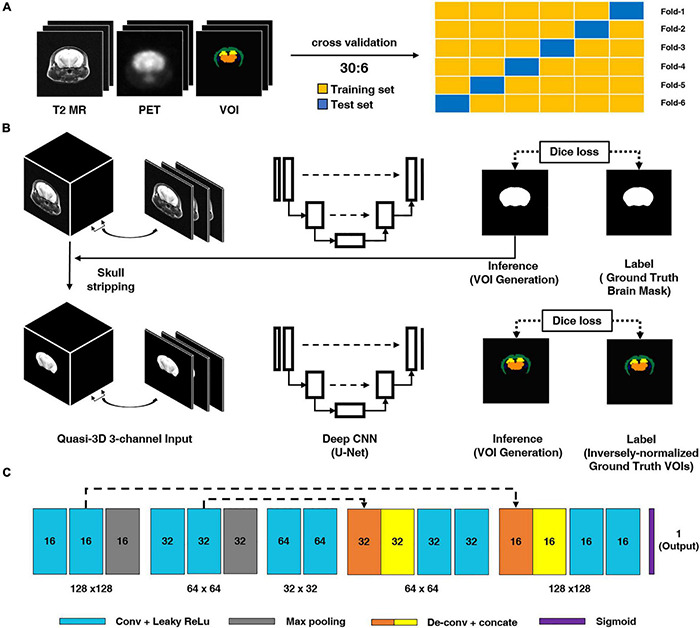
Schematic diagram of the proposed deep CNN model. **(A)** We conducted six-fold crossvalidation for training and test set separation. **(B)** Brain parenchymal mask generation deep CNN. For model training, T2-weighted magnetic resonance (T2 MR) imaging was used as an input and manually traced brain mask was used as a label. **(B)** Inversely normalized volumes-of-interest (iVOIs) in individual brain space generation through deep CNN. For training the deep CNN model, skull-stripped T2 MR was used as the model input and iVOI template was used as the VOIs in individual space (the label for the model). **(C)** Deep CNN (i.e., U-Net) structure used in this study. The light blue box represents a 3 × 3 convolution and a leaky ReLU followed by max pooling, expressed in gray boxes. The orange and yellow boxes denote the 2 × 2 deconvolution and concatenation operations to increase the accuracy of localization. The dash arrow represents the copying skip connections. The dash arrow represents the copying skip connections. deep CNN, deep convolutional neural network.

A CNN is a DL model that enables training while maintaining spatial information and thus allows efficient learning with a lighter architecture than a conventional neural network architecture, that is, fully connected network. For image feature extraction in a CNN, the filters (also known as kernels) in the convolution layers transform the input images into convolution-based filtered output images, termed feature maps. F represents a feature map and is calculated using the following equation:


$$\text{F}\left[m,n\right]=\left(I*k\right)\left[m,n\right]=\sum_{i}\sum_{j}k\left[i,j\right]I\left[m-i,n-j\right]$$

where *I* represents the input image, *k* represents a kernel, and *m* and *n* represent the index of the rows and columns of the resulting matrix, respectively. For each convolution layer, the shape of the output data is changed according to the filter size, stride, and max pooling size and if zero-padding is applied or not. In this study, we used a quasi-3D U-Net for the deep CNN architecture and an input comprising three consecutive slices for the T2 MR images to generate a brain mask for skull-stripping and an iVOI template in an individual space, as illustrated in Figure 1. Specifically, we used three consecutive slice-based multichannel inputs for the generation of each VOI slice; this 2D-like approach can generate as large amount of training data, as a 2D approach (see Supplementary Material for the comparison of the results of the quasi-3D and 2D approaches). We believe that such an approach can additionally overcome the limitations of the small amounts of mouse data. Moreover, the consecutive slice-based information allows the U-Net architecture to generate a highly continuous VOI by referring to the information of contiguous slices. U-Net consists of an end-to-end CNN architecture using a contracting path of the images and an expanding path for localization and residual learning of images to output layers. The contraction path consists of a 3 × 3 convolution followed by a 2 × 2 max pooling using a leaky rectified linear unit (leaky ReLU) as the activation function. The expansion path consists of a 2 × 2 deconvolution for upsampling and a 3 × 3 convolution using a leaky ReLU after concatenating with the context captured in the contraction path to increase the accuracy of localization. To train a DL CNN for skull-stripping mask generation, the abovementioned consecutive axial slices of T2 MR images were used as multichannel input, and manually traced binary brain masks were used as labels. A similar qausi-3D approach was also used in the training of iVOI template generation model, where an iVOI template in an individual space was used as the label. As the U-Net input, the skull-stripping mask generation model and the iVOI template generation model used 70 slices of transaxial MR and the corresponding skull-stripped MR, respectively, and the input dimension was 70 slices × N, 128, 128, 3 channel, where N is the number of mice used in the training set.

To overcome the small amount of data available for training set, data augmentation was performed through shift, rotation, and shear transformations. We used a Dice loss function and an adaptive moment estimation (Adam) optimizer to fit deep CNN parameters. The training was performed with an initial learning rate of 1e-5 with 25 batches. Our deep CNN model was implemented using Keras (version 2.2.4)-based code in the Python programming language with a backbone of TensorFlow (version 1.12.0) running on a GeForce NVIDIA GTX 1080 GPU and an Intel^®^ Xeon^®^ E5-2640 CPU. To regularize the generated mask by the deep CNN, we conducted postprocessing in several steps, different from other studies that used complicated postprocessing methods such as graph cuts (Jimenez-Carretero et al., 2019; Kim et al., 2020; Hu et al., 2021). First, the mask predicted by DL was converted into a binary mask, and erosion and dilation were sequentially performed (also known as the “open” operator) to remove noisy false positives (FPs). Subsequently, 3D-connected component analysis-based kill-islands and fill-holes were conducted to reduce FPs and false negatives (FNs), respectively.

### Performance Evaluation

To avoid overfitting issue and also to increase the generalization ability of the deep CNN, we conducted six-fold crossvalidation for training and test set separation. Of a total 36 samples obtained by 2 scans before and after treatment in 18 mice, 30 samples (15 mice × 2 scans) were used as the training set. The remaining 6 samples (3 mice × 2 scans) were used as the testing set in each fold of the training or test set pair to ensure that the slices of the same mouse are not split as training or test samples. To assess the concordance between predicted and label masks, we measured the Dice similarity coefficient (DSC), average symmetric surface distance (ASSD), sensitivity (SEN), and positive predictive value (PPV). DSC is the most representative indicator used for image segmentation evaluation. It directly compares the results of the two image segmentations, which indicate their similarity. The average symmetrical surface distance is the average of all distances from a point at the boundary of the machine segment region to the boundary of the ground truth. The SEN is defined as the proportion of true positive (TP) results among TP and FN. The SPE is defined as the proportion of true negative (TN) among TN and FP. PPV is defined as the proportion of TP among TP and FP. Formulas are provided for the five methods as follows, where P is the predicted mask of our network, and G is the label used in deep CNN (See following equations for the details).


$$DiceSimilarityCoefficient=\frac{2\left|P\cap G\right|}{\left|P\right|+\left|G\right|},$$

$$AverageSymmetricSurfaceDistance=\frac{\sum_{m\in \partial \left(M\right)}\underset{g\in \partial \left(G\right)}{min}||p-g||\sum_{g\in \partial \left(G\right)}\underset{m\in \partial \left(M\right)}{min}||g-p||}{\left|\partial \left(P\right)\right|+\left|\partial \left(G\right)\right|}$$

$$Sensitivity=\frac{TP}{\text{TP}+\text{FN}},$$

$$Specificity=\frac{\text{TN}}{\text{TN}+\mathrm{FP}},$$

$$PositivePrecitiveValue=\frac{\text{TP}}{\text{TP}+\text{FP}}.$$

Standardized uptake value (SUV), the most representative and simplest method of determining activities in PET images, is widely used for PET (semi)quantitative analysis. SUV can be calculated by the equation below.


$$\text{SUV}\left(\text{t}\right)=\frac{C_{PET}\left(t\right)}{ID/BW},$$

where C_*PET*_(t) is the radioactivity measured from an image acquired at the time t, ID is the injected dose at t = 0, and BW is the animal’s body weight. The ratio of the SUV from target region and reference region within the same PET image is commonly called standardized uptake value ratio (SUVR). In ^18^F-FDG PET, a PET image is obtained based on glucose uptake metabolism after sufficient time elapses, following the injection of the tracer into the body. Then, SUVR was calculated by dividing target regions of glucose metabolism depending on the disease, with constant glucose uptake regions at all times regardless of the disease. In this study, the SUVR evaluation was conducted on four major regions: cortex, hippocampus, thalamus, and stratum. We chose the cerebellum as a reference region, which seems to be free of plaques in the AD mouse model.


$$\text{SUVR}=\frac{SUV_{target}}{SUV_{reference}}=\frac{C_{img,target}}{C_{img,reference}}.$$

To assess our DL-based method, mean count and SUVR were calculated by the following three different methods: DL-generated mask (VOI_DL_), DL label mask (i.e., inversely normalized template (ground-truth) VOIs, VOI_iGT_), and template-based ground-truth VOI (VOI_GT_). Then, correlation analysis was conducted between these methods. In addition, we compared percentage change (% change) in SUVR before and after the treatment of 18 mice obtained by the abovementioned three masks by qualitative assessment and quantitative assessment by conducting paired or unpaired two-sample *t*-tests.

## Results

### Deep Convolutional Neural Network for Automatic Generation of Brain Mask for Skull-Stripping

**FIGURE 2:**
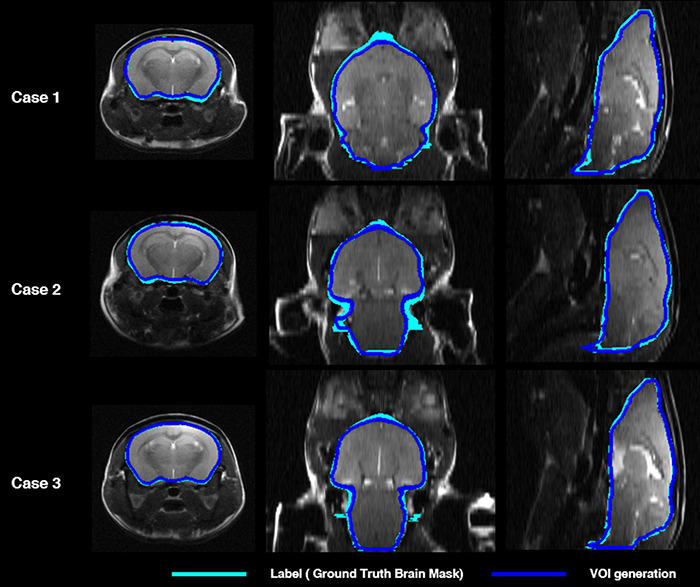
Comparison of mouse brain parenchyma mask contours between manually traced brain mask (light blue) and deep CNN-predicted brain mask (blue) in three cases. The left shows the axial plane, the middle the coronal plane, and the right shows the sagittal plane. deep CNN, deep convolutional neural network.

Figure 2 shows the comparisons in the brain skull-stripping mask generated by the proposed deep CNN (i.e., U-Net) (blue contour) and a manually traced brain mask (light blue contour) in three mice. All three mice showed high visual consistency without significant differences.

Table 1 shows the concordance of brain masks generated by a deep CNN and manually traced masks in terms of the DSC, ASSD, SEN, SPE, and PPV. Mean DSC reached 0.97, mean ASSD was less than 0.01 mm, and mean SEN reached 0.92. In addition, both mean SPE and mean PPV were greater than 0.99. In summary, there was no significant difference between the brain mask generated by our DL-based method and the manually traced mask in the six-fold crossvalidation.

**TABLE 1:** Our unified deep neural network assessment through the mean and standard deviation of Dice similarity coefficient (DSC), average symmetric surface distance (ASSD) (mm), sensitivity (SEN), and positive predictive value (PPV) between DL labels and DL-generated brain masks and inversely normalized target VOIs (cortex, hippocampus, striatum, thalamus, and cerebellum) in six-fold crossvalidation.

	N-fold	Brain mask	Cortex	Hippocampus	Striatum	Thalamus	Cerebellum
DSC	Fold-1	0.96 ± 0.01	0.78 ± 0.02	0.68 ± 0.2	0.62 ± 0.18	0.76 ± 0.12	0.66 ± 0.05
	Fold-2	0.95 ± 0.01	0.78 ± 0.01	0.76 ± 0.07	0.72 ± 0.03	0.72 ± 0.11	0.69 ± 0.04
	Fold-3	0.96 ± 0.01	0.79 ± 0.01	0.66 ± 0.1	0.66 ± 0.04	0.74 ± 0.11	0.7 ± 0.01
	Fold-4	0.98 ± 0.02	0.77 ± 0.04	0.74 ± 0.08	0.74 ± 0.11	0.78 ± 0.03	0.72 ± 0.03
	Fold-5	0.96 ± 0.01	0.77 ± 0.02	0.68 ± 0.13	0.75 ± 0.08	0.77 ± 0.08	0.68 ± 0.02
	Fold-6	0.98 ± 0.02	0.75 ± 0.02	0.61 ± 0.13	0.59 ± 0.06	0.76 ± 0.12	0.69 ± 0.02
	Total	0.97 ± 0.01	0.77 ± 0.02	0.69 ± 0.14	0.68 ± 0.13	0.76 ± 0.01	0.71 ± 0.03
ASSD (mm)	Fold-1	0.01 ± 0.00	0.08 ± 0.01	0.11 ± 0.06	0.2 ± 0.13	0.12 ± 0.03	0.12 ± 0.01
	Fold-2	0.01 ± 0.00	0.09 ± 0.0	0.12 ± 0.06	0.08 ± 0.01	0.09 ± 0.01	0.09 ± 0.01
	Fold-3	0.01 ± 0.0	0.08 ± 0.0	0.11 ± 0.04	0.1 ± 0.01	0.02 ± 0.04	0.11 ± 0.01
	Fold-4	0.02 ± 0.01	0.09 ± 0.01	0.12 ± 0.07	0.11 ± 0.04	0.04 ± 0.02	0.05 ± 0.01
	Fold-5	0.01 ± 0.00	0.09 ± 0.01	0.12 ± 0.08	0.1 ± 0.02	0.12 ± 0.01	0.07 ± 0.01
	Fold-6	0.01 ± 0.0	0.09 ± 0.0	0.16 ± 0.08	0.11 ± 0.02	0.22 ± 0.08	0.15 ± 0.02
	Total	0.01 ± 0.00	0.17 ± 0.0	0.12 ± 0.07	0.12 ± 0.06	0.1 ± 0.00	0.01 ± 0.00
SEN	Fold-1	0.91 ± 0.02	0.76 ± 0.05	0.67 ± 0.29	0.6 ± 0.15	0.68 ± 0.02	0.61 ± 0.05
	Fold-2	0.94 ± 0.02	0.81 ± 0.04	0.66 ± 0.11	0.6 ± 0.06	0.65 ± 0.05	0.64 ± 0.12
	Fold-3	0.93 ± 0.02	0.77 ± 0.03	0.53 ± 0.19	0.65 ± 0.08	0.69 ± 0.05	0.71 ± 0.14
	Fold-4	0.97 ± 0.02	0.73 ± 0.06	0.7 ± 0.15	0.65 ± 0.14	0.69 ± 0.01	0.75 ± 0.05
	Fold-5	0.88 ± 0.04	0.78 ± 0.07	0.55 ± 0.22	0.56 ± 0.12	0.66 ± 0.12	0.68 ± 0.08
	Fold-6	0.87 ± 0.01	0.72 ± 0.07	0.52 ± 0.25	0.58 ± 0.08	0.64 ± 0.02	0.72 ± 0.07
	Total	0.92 ± 0.02	0.76 ± 0.06	0.61 ± 0.22	0.61 ± 0.16	0.67 ± 0.05	0.69 ± 0.09
SPE	Fold-1	1.0 ± 0.0	1.0 ± 0.0	1.0 ± 0.0	1.0 ± 0.0	0.99 ± 0.0	0.99 ± 0.0
	Fold-2	1.0 ± 0.0	1.0 ± 0.0	1.0 ± 0.0	1.0 ± 0.0	0.99 ± 0.0	0.99 ± 0.00
	Fold-3	1.0 ± 0.0	1.0 ± 0.0	1.0 ± 0.0	1.0 ± 0.0	0.99 ± 0.0	1.0 ± 0.0
	Fold-4	1.0 ± 0.0	1.0 ± 0.0	1.0 ± 0.0	1.0 ± 0.0	1.0 ± 0.0	1.0 ± 0.0
	Fold-5	1.0 ± 0.0	1.0 ± 0.0	1.0 ± 0.0	1.0 ± 0.0	1.0 ± 0.0	1.0 ± 0.0
	Fold-6	1.0 ± 0.0	1.0 ± 0.0	1.0 ± 0.0	1.0 ± 0.0	1.0 ± 0.0	1.0 ± 0.0
	Total	1.0 ± 0.0	1.0 ± 0.0	1.0 ± 0.0	1.0 ± 0.0	0.99 ± 0.0	0.99 ± 0.0
PPV	Fold-1	0.98 ± 0.01	0.8 ± 0.03	0.65 ± 0.14	0.88 ± 0.04	0.73 ± 0.01	0.68 ± 0.03
	Fold-2	0.99 ± 0.01	0.76 ± 0.02	0.67 ± 0.06	0.81 ± 0.03	0.8 ± 0.06	0.78 ± 0.07
	Fold-3	0.99 ± 0.0	0.8 ± 0.02	0.67 ± 0.09	0.82 ± 0.05	0.78 ± 0.03	0.71 ± 0.03
	Fold-4	0.99 ± 0.0	0.81 ± 0.01	0.6 ± 0.03	0.8 ± 0.06	0.82 ± 0.03	0.65 ± 0.02
	Fold-5	0.99 ± 0.01	0.76 ± 0.03	0.72 ± 0.11	0.8 ± 0.04	0.77 ± 0.03	0.74 ± 0.03
	Fold-6	0.99 ± 0.0	0.8 ± 0.05	0.58 ± 0.07	0.76 ± 0.01	0.78 ± 0.02	0.68 ± 0.04
	Total	0.99 ± 0.0	0.79 ± 0.04	0.65 ± 0.1	0.81 ± 0.06	0.78 ± 0.03	0.71 ± 0.01

### Deep Convolutional Neural Network for Automatic Generation of Inversely Normalized VOI Template in Individual Brain Space

We conducted a qualitative visual assessment and quantitative assessment of VOI_DL_ and VOI_iGT_ to evaluate the performance of the proposed deep CNN model. In addition, we conducted a correlation analysis between mean count in each VOI obtained using VOI_DL_, VOI_iGT_, and VOI_GT_ and correlation analysis between each mean SUVR using the abovementioned VOI method. In addition, % change of SUVR between PET images before and after treatment was analyzed through a two-sample *t*-test for each VOI mask.

**FIGURE 3:**
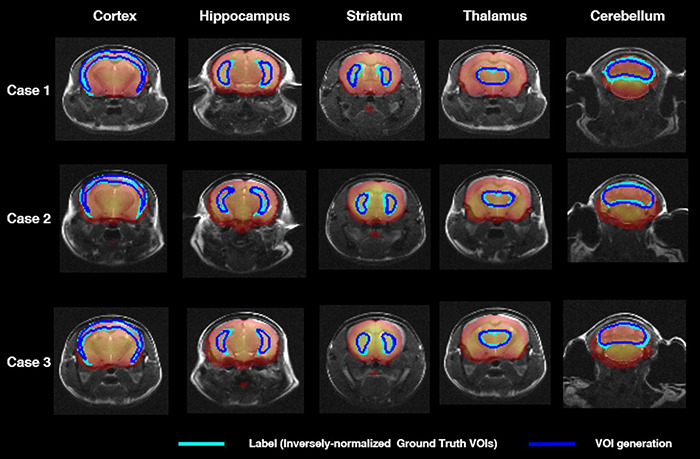
Segmentation comparison of target VOIs (i.e., cortex, hippocampus, striatum, thalamus, cerebellum) in axial plane of three mice. The light blue contour represents deep learning label masks which are iVOI templates. The blue contour represents an iVOI template in an individual space generated by the proposed deep CNN. iVOI, inversely normalized VOI; deep CNN, deep convolutional neural network.

Figure 3 shows axial slices of VOI_DL_ (blue contour) and VOI_iGT_ (light blue contour) in target VOIs (cortex, hippocampus, stratum, thalamus, and cerebellum). There was no significant difference between VOI_DL_ and VOI_iGT_ in cortex, cerebellum, and thalamus and also in the relatively small areas of the hippocampus and striatum.

In Table 1, VOI_DL_ and VOI_iGT_ of 36 mice in target VOIs (cortex, hippocampus, striatum, thalamus, and cerebellum) were evaluated by calculating the average of DSC, ASSD, SEN, SPE, and PPV. The DSC for each target VOI was 0.77, 0.69, 0.68, 0.76, 0.69, the ASSD was 0.17, 0.12, 0.12, 0.1, and 0.01 mm, SEN was 0.76, 0.61, 0.61, 0.67, and 0.69, SPE was 1.0, 1.0, 1.0, 0.99, and 0.99, and PPV was 9, 0.65, 0.81, 0.78, and 0.71, respectively, which indicates that our DL model generated target VOIs well.

**FIGURE 4:**
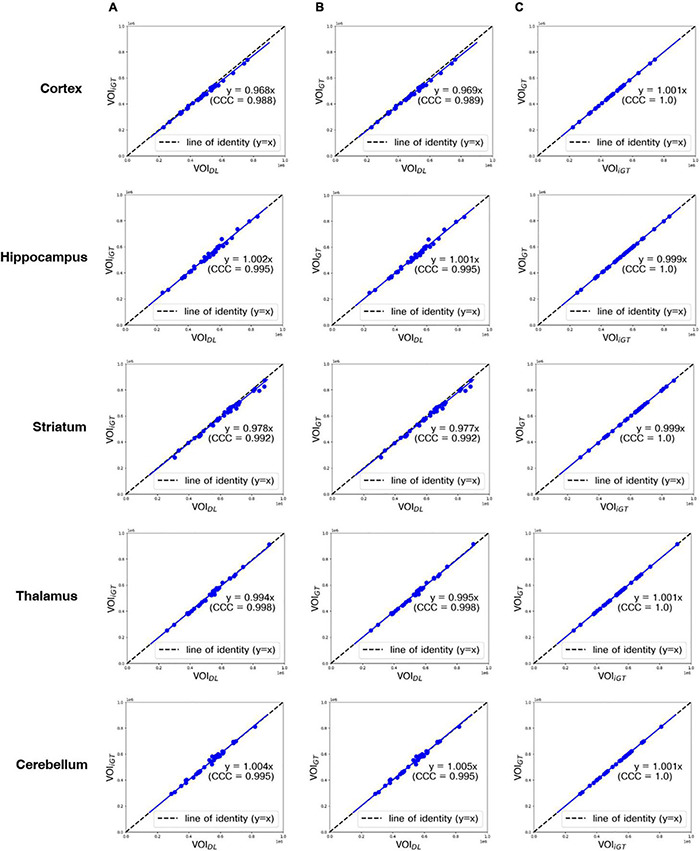
Correlation analysis between all mean counts (blue dot) obtained using VOI_DL_, VOI_iGT_, and VOI_GT_ in all target VOIs from the first to the fifth row (cortex, hippocampus, striatum, thalamus, and cerebellum, respectively). CCC represents a measure of reliability based on covariation and correspondence. The line of identity (dashed line) is depicted as a reference line. Cortex, hippocampus, striatum, thalamus, and cerebellum are represented sequentially from the top row. **(A)** Correlation analysis between each mean count obtained using VOI_DL_ and VOI_*iGT*_. **(B)** Correlation analysis between each mean count obtained using VOI_DL_ and VOI_GT_. **(C)** Correlation analysis between each mean count obtained using VOI_iGT_ and VOI_GT_ VOI_*DL*,_ deep-learning generated-VOI; VOI_iGT_, deep learning (inverse-normalized ground-truth) label VOI; VOI_GT,_ template-based ground-truth VOI; CCC, concordance correlation coefficient.

**FIGURE 5:**
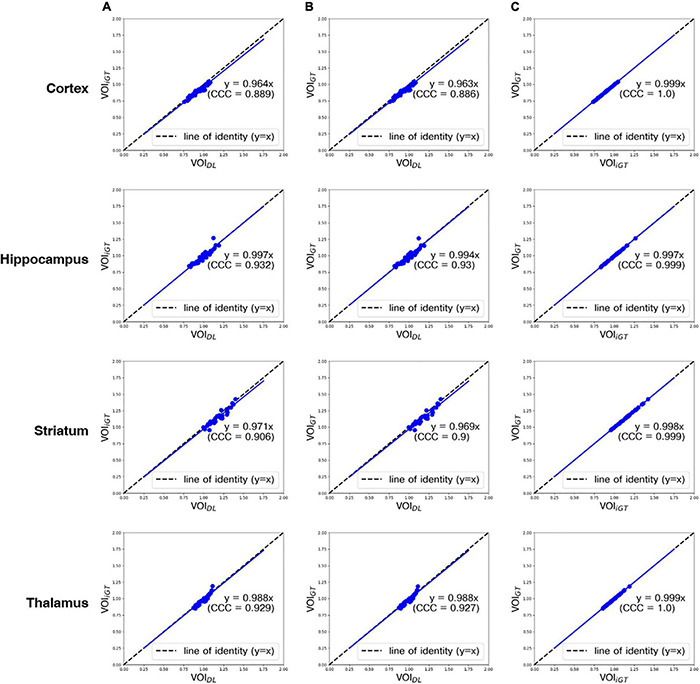
Correlation analysis between all mean SUVRs (blue dot) obtained using VOI_DL_, VOI_iGT_, and VOI_GT_ in all target VOIs from the first to the fourth rows (cortex, hippocampus, striatum, and thalamus, respectively). Concordance correlation coefficient represents a measure of reliability based on covariation and correspondence. The line of identity (dashed line) is depicted as a reference line. Cortex, hippocampus, striatum, and thalamus are represented sequentially from the top row. **(A)** Correlation analysis between mean SUVRs obtained using VOI_DL_ and VOI_*iGT*_. **(B)** Correlation analysis between mean SUVRs obtained using VOI_DL_ and VOI_GT_. **(C)** Correlation analysis between each mean SUVRs obtained using VOI_iGT_ and VOI_GT_ VOI_*DL*,_ deep learning generated VOI; VOI_iGT_, deep learning label VOI; VOI_GT,_ template-based VOI; CCC, concordance correlation coefficient.

In Figure 4, the mean counts obtained by VOI_DL_, VOI_iGT_, and VOI_GT_ were significantly (*p* < 0.001) correlated with one another in all target VOIs. In addition, as shown in Figure 4B, the mean count obtained by VOI_GT_ in template space tended to be slightly underestimated compared to the mean count obtained by VOI_DL_ in individual space. Furthermore, we conducted a correlation analysis between each mean SUVR obtained using VOI_DL_, VOI_iGT_, and VOI_GT_ in all target VOIs (Figure 5). For SUVR analysis, cerebellum was used as a reference region, that is, SUV (or mean count) of target regions was normalized by that of cerebellum. In all target VOIs, mean SUVR obtained through VOI_DL_, VOI_iGT_, and VOI_GT_ was significantly (*p* < 0.001) correlated with one another. As shown in Figure 5B, in the hippocampus and thalamus, the mean SUVR obtained by VOI_GT_ in template space and the VOI_DL_ in individual space was almost identical, whereas in the cortex and striatum, the mean SUVR obtained by VOI_GT_ was underestimated compared to the mean SUVR obtained by VOI_DL_.

**FIGURE 6:**
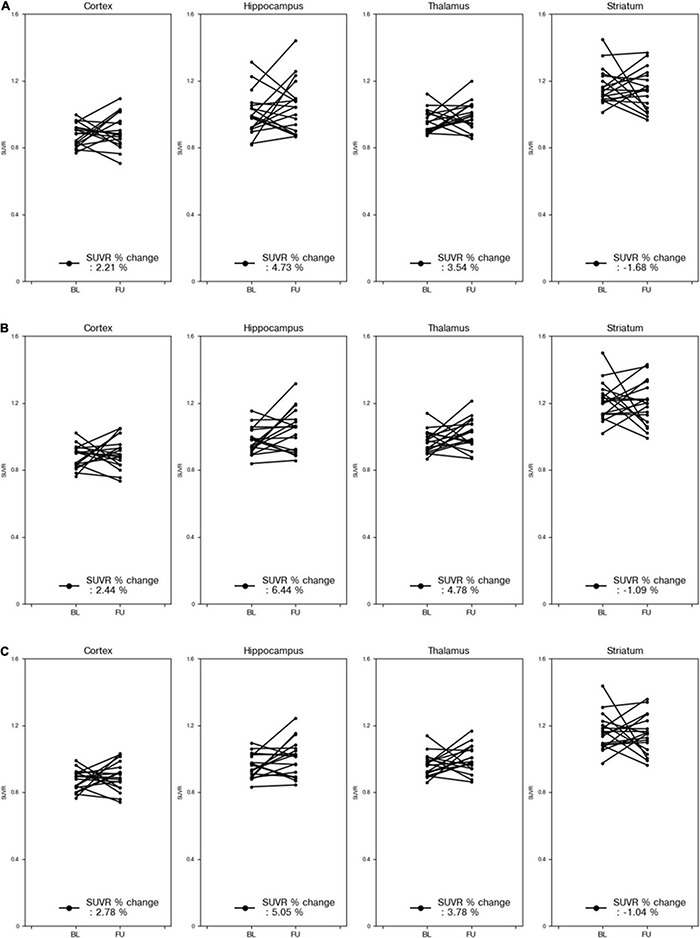
% changes in SUVR for each part of 18 mice before and after treatment. The reference area of the SUVR was the cerebellum. Percent change in SUVR obtained using VOI_DL_ **(A)**. Percent change in SUVR obtained using VOI_iGT_ **(B)**. Percent change in SUVR obtained using VOI_GT_ **(C)**. Results for cortex, hippocampus, striatum, and thalamus are presented sequentially from the left column. VOI_*DL*,_ deep learning generated VOI; VOI_iGT_, deep learning label VOI; VOI_GT,_ template-based VOI; baseline (BL—before treatment); follow-up (FU—after treatment).

Figure 6 compares the % changes of the SUVRs in each VOI before and after treatment using VOI_DL_, VOI_iGT_, and VOI_GT_ for 18 mice. Regardless of the three types of masks, there was no significant difference between the mean SUVR % changes obtained by each mask. Moreover, the degrees of increase in the mean SUVRs in the cortex, hippocampus, and thalamus were similar, and the degree of decrease in those in the striatum was similar. In addition, we did not find significant differences in the SUVR% changes of all target VOIs before and after treatment of three methods (DL-based method and SN/iSN-based conventional methods) by both paired and unpaired two-sample *t*-tests (*p* > 0.3 and *p* > 0.8, respectively).

## Discussion and Conclusion

Spatial normalization of individual brain PET images onto standard anatomical spaces is required for objective statistical evaluation (Feo and Giove, 2019). However, several steps of the preprocessing were semiautomatic, which causes time-consuming problems and inter- and intrarater problem. A precise SN method through DL has also been devised and has recently attracted considerable attention. However, despite the use of a complicate network of structures, the method still has difficulty in performing complete SN from affine transformation to non-linear transformation (Alvén et al., 2019), and an additional SN process is required to generate a PET pseudotemplate (Kang et al., 2018) or a method of generating MR from a PET image (Choi et al., 2018). In addition, many studies have considered the use of iSN for PET quantitative analysis (Kim et al., 2010, 2015; Cho et al., 2014). Inspired by this, we generated an iVOI template to reformulate the complicated and difficult-to-implement SN problem into a relatively simpler problem of VOI segmentation for PET quantification. Overall, we have devised a unified CNN framework for brain mask generation to skull-stripping and generate an iVOI template in individual space to perform precise PET quantification without any additional efforts of skull-stripping and SN.

With regard to DL for brain parenchyma segmentation, as shown in Figure 2, manually traced brain mask contours (light blue) and DL-based contours (blue) were almost identical in visual assessment. Moreover, DSC reached 0.97, ASSD was less than 0.01 mm, and SEN and PPV were higher than 0.92 and 0.99 (Table 1) compared between these two masks, confirming good agreement from a quantitative perspective as well. With regard to DL for iVOI template segmentation, mean count and SUVR obtained by VOI_iGT_ and VOI_DL_ were significantly (*p* < 0.001) correlated with each other in all target VOIs (Figures 3A, 4A). In addition, each of the mean count and SUVR obtained with template-based VOI (i.e., VOI_GT_) and individual space VOI (i.e., VOI_DL_, VOI_iGT_) showed a significant (*p* < 0.001) correlation between each VOI methods (Figures 3B,C, 4B,C). However, the mean count and SUVR obtained by VOI_GT_ tended to be slightly underestimated compared to that obtained by VOI_iGT_ and VOI_DL_ in hippocampus and striatum. We believe that our method can avoid image intensity degradation problems that may occur in preprocessing, including SN. Consequently, the % change of SUVR in the target VOI showed the same tendency in each of the three methods. In summary, the proposed DL-based method using an iVOI template in individual space that does not require SN has been developed as new method of PET quantitative assessment, avoiding variation between intra- and interrater that can occur in the process of drawing a brain mask or in the preprocessing. In addition, we showed a comparable level of segmentation performance using lighter neural network structures (i.e., CNNs with fewer training parameters—fewer channels and simple convolutional blocks) as compared to conventional mouse brain studies (Hsu et al., 2020; De Feo et al., 2021). Furthermore, our DL-based iVOI method has reformulated the PET SN problem for precise PET quantification, which remains challenging despite using a complicate network that requires a considerable parameter estimation (Choi et al., 2018; Kang et al., 2018; Alvén et al., 2019), with an easy-to-handle image generation method for target VOIs using inversely normalized template VOIs.

This study involves several limitations. First, we only had small number of data, consisting of 18 mice. Although the data were inflated using consecutive axial slices of T2 MR as multichannels input of the deep CNN and using data augmentation, this was not sufficient. Second, because this study was carried out with only in-house mice with a specific disease (i.e., Alzheimer’s disease), there is a possibility that the trained model may be specialized to our data. Therefore, as in study by De Feo et al. (2021), model validation should be warranted with more data and various types of mice in the near future. Third, our method is dependent on MR images because we generated iVOI templates using parameters based on SN of MR images onto MR templates in the process of generating label used for deep CNN training. However, corresponding MR images are not always available in PET image analyses. In this regard, we should consider further studies for generating iVOI templates only with PET images. Finally, one of the strengths of this study is that because we used a PET/MRI system registration of PET, MR is straightforward in nature compared to existing methods. Nonetheless, we believe that our proposed approach may be readily applied to ordinary PET or PET/CT scanners with additional MR images as well. In this regard, a further study on a more unified deep CNN with PET input and iVOI defined by MR should be warranted in the near future.

In conclusion, we proposed a unified deep CNN-based model that can generate mouse brain parenchyma masks and iVOI templates in individual brain space without any effort for skull-stripping and SN. Through qualitative and quantitative evaluations, our proposed model has shown identical quantification of regional glucose metabolism (i.e., mean SUV of each VOI) forming line of identity between ground truth (conventional template) methods-based mean SUV/SUVR and DL-based mean SUV/SUVR (Figures 4, 5, respectively) and also concordant patterns of mean SUVR treatment-induced change between ground truth and DL methods. These results show that the proposed approach comprises a new method for PET image analysis by reformulating the SN problem, which has been difficult to implement despite of recent advances in DL techniques, into a segmentation problem using iVOI template generation in an individual brain space.

## Data Availability Statement

The datasets presented in this article are not readily available because because this study cohort is not open for public use. Requests to access the datasets should be directed to JO, jungsu.oh@gmail.com.

## Ethics Statement

The animal study was reviewed and approved by the Institutional Animal Care and Use Committee (IACUC), Asan Institute for Life Sciences, Asan Medical Center.

## Author Contributions

SS, S-JK, and JO contributed to study conceptualization, data acquisition, data analysis, data interpretation, writing, and editing of the manuscript. JC, S-YK, and SO contributed to data acquisition and data interpretation. SJ and JK contributed to study conceptualization, data interpretation, and editing of the manuscript. All authors contributed to the article and approved the submitted version.

## Conflict of Interest

The authors declare that the research was conducted in the absence of any commercial or financial relationships that could be construed as a potential conflict of interest.

## Publisher’s Note

All claims expressed in this article are solely those of the authors and do not necessarily represent those of their affiliated organizations, or those of the publisher, the editors and the reviewers. Any product that may be evaluated in this article, or claim that may be made by its manufacturer, is not guaranteed or endorsed by the publisher.
