# Supplementary material for: Unified Deep Learning-Based Mouse Brain MR Segmentation: Template-Based Individual Brain Positron Emission Tomography Volumes-of-Interest Generation Without Spatial Normalization in Mouse Alzheimer Model
Source: Front Aging Neurosci. 2022 Mar 4;14:807903. doi: 10.3389/fnagi.2022.807903 (PMC8931825; doi:10.3389/fnagi.2022.807903)
Supplement: Supplementary file 1 [file Image_1.pdf]

Supplementary Material

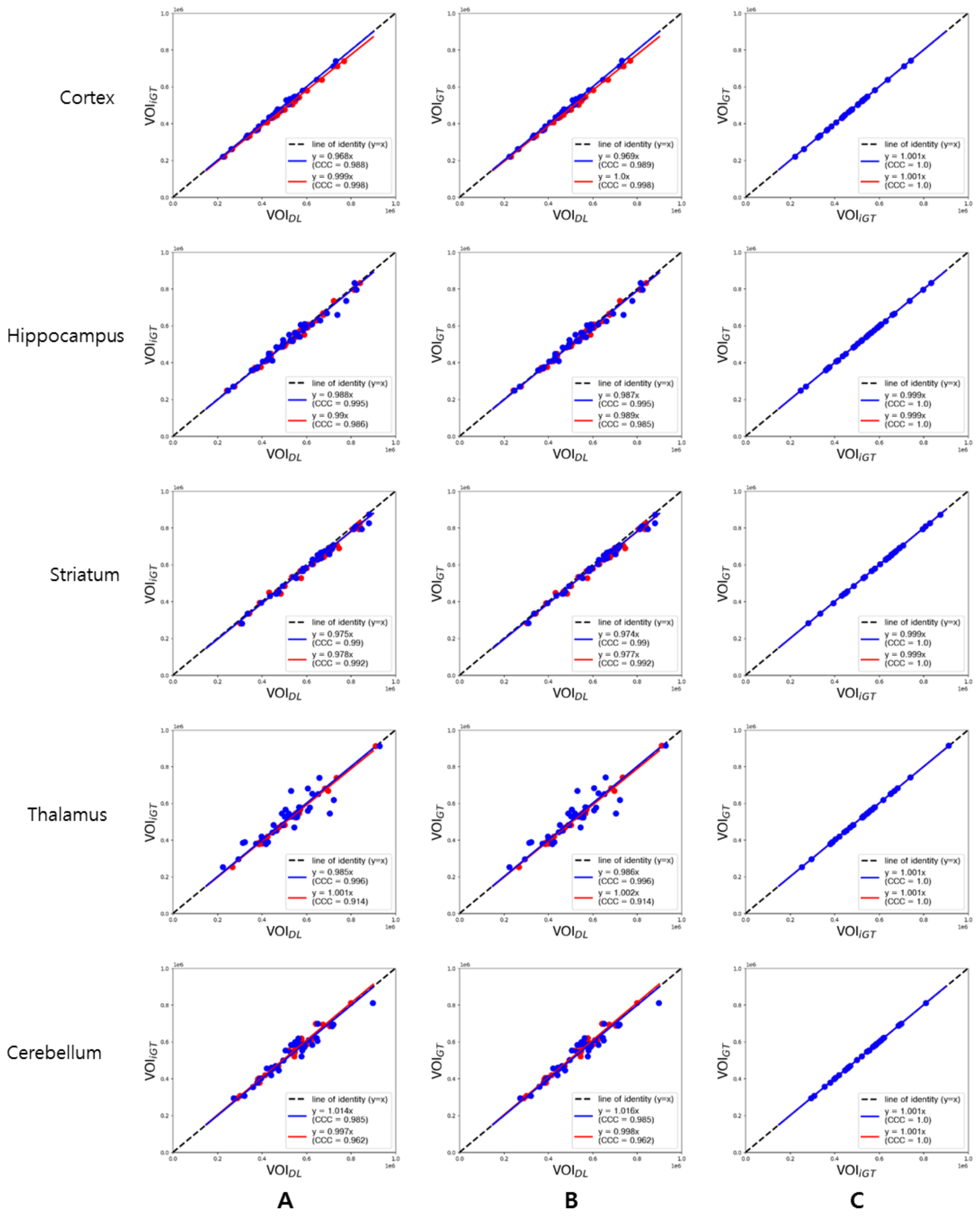

Supplementary Figure 1. Comparison of SUV quantification results between 3D U-Net architecture (blue lines and dots, 6-fold cross-validation with quasi using three consecutive slices) and 2D U-Net architecture (red lines and dots, 6-fold cross-validation).
